# Universal limiting shape of worn profile under multiple-mode fretting conditions: theory and experimental evidence

**DOI:** 10.1038/srep23231

**Published:** 2016-03-16

**Authors:** Andrey I. Dmitriev, Lars B. Voll, Sergey G. Psakhie, Valentin L. Popov

**Affiliations:** 1 Institute of Strength Physics and Materials Science SB RAS, 634055, Tomsk, Russia; 2 National Research Tomsk Polytechnic University, 634050, Tomsk, Russia; 3 National Research Tomsk State University, 634050, Tomsk, Russia; 4 Berlin University of Technology, 10623 Berlin, Germany

## Abstract

We consider multiple-mode fretting wear in a frictional contact of elastic bodies subjected to a small-amplitude oscillation, which may include in-plane and out-of-plane translation, torsion and tilting (“periodic rolling”). While the detailed kinetics of wear depends on the particular loading history and wear mechanism, the final worn shape, under some additional conditions, occurs to be universal for all types and loading and wear mechanisms. This universal form is determined solely by the radius of the permanent stick region and the maximum indentation depth during the loading cycle. We provide experimental evidence for the correctness of the theoretically predicted limiting shape. The existence of the universal limiting shape can be used for designing joints which are resistant to fretting wear.

Fretting wear occurs almost inevitably in frictional contacts subjected to small amplitude alternating loads (mechanical vibration or thermal cycling). Even if the oscillating loads are well below the static friction limit, they generally will lead to a repeated slip in the boundary region of the contact; this may cause progressive changing of the local form of the contacting partners and fretting fatigue. The problem of fretting wear attracts much attention because of its technological importance for such applications as wear in steam generator tubes^1^,^2^, biomedical implants^3^,^4^, electrical contacts^5^,^6^, corrosion and materials degradation^7^, dovetail blade roots of gas turbines^8^,^9^ and many others. It remains an object of intensive experimental and theoretical investigation as there are still several open problems, especially concerning wear under complex load histories as well as development of principles of “anti-fretting design”.

In a general case, the relative movement of contacting bodies can include tangential oscillations in the two in-plane directions, normal oscillations, torsion around an axis normal to the contact plane as well as tilting (rolling) around two horizontal axes. Correspondingly, depending on the direction of relative displacement or rotation of bodies in contact, different fretting “modes” can be distinguished: tangential, radial, rolling and torsional fretting, while the list can be continued. Up to now, most research was focused on fretting wear in tangential mode^10^,^11^,^12^; there are only few reports on other single-mode fretting regimes as torsional^13^ or rotational^14^,^15^, even less on dual-motion fretting^4^,^5^,^16^ and practically no studies on multiple-mode fretting. In the present paper, we consider the general case of superimposed oscillations in all three possible directions as well as rotation around three possible axes. We will discuss some cases of kinetics of the progressive fretting wear; however, our main attention is focused on the *limiting shape* of the worn surfaces (if this limiting shape does exist). We will show that for system, in which the normal and tangential contact problems can be considered as uncoupled, this limiting shape does not depend on particular mode of loading.

In the case of two-dimensional contacts and uni-directional tangential oscillations, Ciavarella and Hills^17^ argued that the wear rate in fretting process slows down as the wear process goes on. This is due to continuous decrease of pressure in the worn region and increase of pressure in the initial stick region. If the wear occurs continuously (without breaking out of larger wear debris) and the worn material is continuously transported out of the contact zone, the worn profile will tend to some limiting shape in which no further wear occurs. The existence of the limiting shape for three-dimensional axis-symmetric contacts was proven in a recent paper^18^, where a procedure for the analytical calculation of the limiting profile has also been described. In the present paper, we will show that this limiting shape belongs to a universal class of shapes which are independent of the particular loading mode; this allows formulating a universal principle for “anti-fretting” design. We start with a qualitative discussion of the physical nature of this generality.

## Results

### Universality of the limiting shape in conditions of multiple-mode fretting wear

Let us consider two elastic bodies with an axis-symmetric differential profile 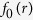, where 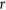 is the in-plane polar radius. The corresponding contact problem can always be equivalently replaced by a contact of an elastic half-space with renormalized elastic properties and a rigid axis-symmetrical indenter. We will only consider the differential worn profile and assume for simplicity that only the rigid profile is worn.

Contrary to the most theoretical studies of fretting, we will not assume any particular wear and friction laws but only make the following very general assumptions about the local wear rate and frictional forces: (a) Wear should only occur in the contact areas with non-vanishing relative displacement of surfaces (thus merely existence of tangential stresses is not enough for initiating the wear process); (b) Wear should occur only in the contact areas with non-vanishing pressure; (c) The law of friction (which does not have necessarily to be the Coulomb law) allows for existence of the region of permanent stick.

**Figure 1:**
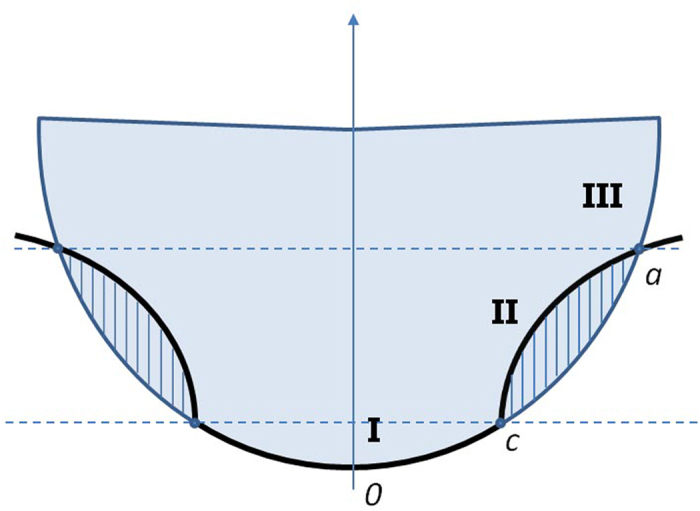
Three regions in the final worn profile. I is the region of permanent stick where no profile changes take place, II is the part, in which no-pressure condition is achieved in the final state (state of “incipient contact”), III is the region of no-contact.

Let us show that already these assumptions determine unambiguously the limiting worn shape. From the existence of a region of permanent stick (assumption (c)) it follows that in this region there will be no wear (assumption (a)), and that the shape of the indenter in this region will coincide with the initial non-worn shape (region I in Fig. 1). Outside of the region of permanent stick, the wear can only vanish if the pressure reduces to zero (assumption (b)). This means that the no-contact condition must be fulfilled. However, as this form has to be achieved *due to wear*, the limiting profile of the indenter must exactly coincide with the form of the *free surface* (no-pressure condition!) produced by the initial indenter shape inside the radius of the permanent stick (region II in Fig. 1). Thus, in the final state, in the region II, the indenter and the elastic half space are in the state of “incipient contact”. Finally, in the region III there is no contact and the indenter retains its unworn shape, as in the region I. The outer radius of worn region, 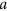, is determined by the intersection of the free surface profile in the region II with the actual non-worn shape of the indenter.

Thus, the limiting shape of the worn profile is determined solely by the solution of the normal contact problem of an indenter having the shape of the initial non-worn profile inside the region of permanent stick; the initial shape outside the region of permanent stick is irrelevant. The limiting shape therefore may only depend on the parameters determining the normal contact solution: the radius 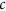 of the permanent stick as well as the maximum indentation depth during the oscillation cycle: exactly in the moment of the maximum indentation the worn surface of the indenter and the free surface of the elastic half-space will “touch” each other (come into “incipient contact”). In this state, small *tangential or rotational* movements of any kind will not change the state of the normal contact and thus will not lead to any wear. The limiting worn shape in the region II therefore does depend on the shape of the profile in the region I but *does not depend on the kind of oscillating loading*. Thus, the shape of the worn profile found in^18^ for the case of single-mode fretting under tangential oscillations is valid for *any* other fretting mode, including arbitrary superposition of tangential and normal oscillations, torsion and tilting. This is of course strictly valid only in the half-space approximation (that is under assumption of small slope of profile, including the worn profile in all points of the contact area). The possible influence of the geometrical non-linearity is not considered here and remains open. However, the experimental investigation strongly supports the results obtained in the half-space-approximation (see below).

### Experimental determination of the limiting shape

**Figure 2:**
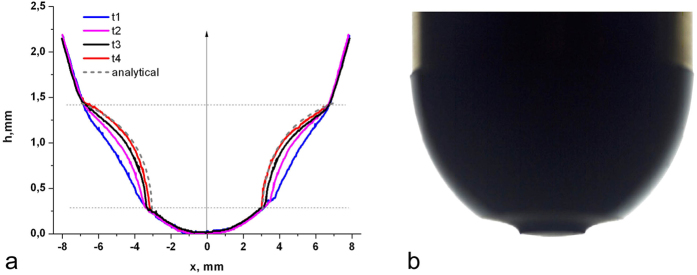
(**a**) The cross section of spherical indenter worn profile obtained experimentally due to *torsional* fretting wear at four consecutive time moments. Note the difference of vertical and horizontal scaling. The theoretical limiting worn shape calculated according to Eq. (3) for the same parameters as in experiment is shown by dashed curve. (**b**) The photo of the experimental indenter in the final state.

To experimentally validate the predicted limiting shape an experimental set up described in the Section “Methods” was designed. As the predicted limiting shape does not depend on the particular fretting mode, the torsional mode was chosen for experimental validation as it can be simply experimentally implemented and controlled. A rubber half-ball was pressed against a rigid abrasive surface and worn due to a periodical torsional loading around the vertical symmetry axis of the ball. The evolution of the worn profile was determined from photographs of the ball at different times Fig. 2a shows the progressive wear after four intervals of 2,160 oscillations. The progress of wear after intervals of oscillation cycles show that the wear is slowing down and the profile tends to some limiting shape as depicted in Fig. 2b. The theoretically predicted limiting shape is shown in Fig. 2a by dashed curves, and it clearly marks the shape to which the real experimental wear shapes are tending.

In the paper^18^, explicit equations for the limiting shape were provided for the case of a sample having initially a parabolic form. Because in our experiments the radius of the permanent stick and the radius of the worn region cannot be considered as small compared with the radius of the ball, we used for the calculation of the limiting shape shown in Fig. 2 the exact ball form:


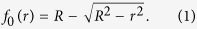


The limiting shape is determined by the Eq. (6) (see Section “Methods”) where the function 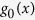 used in this Eq. can be calculated by substituting (1) into (7):


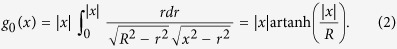


Thus, the explicit form of the limiting shape for the case when the initial shape was a ball with radius 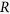 is given by


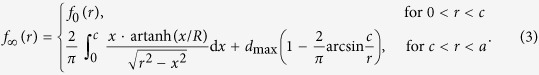


As already discussed above, this shape is determined solely by the initial form (which in this case is characterized by the radius 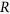 of the ball), the maximum indentation depth 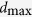 and the radius 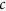 of region of permanent stick. The relation (3) calculated for the values of above three parameters measured in the experiment shown in Fig. 2 are presented in this Figure with dashed line. The measured shape after 8640 oscillation cycles agrees amazingly well with the predicted limiting shape.

**Figure 3:**
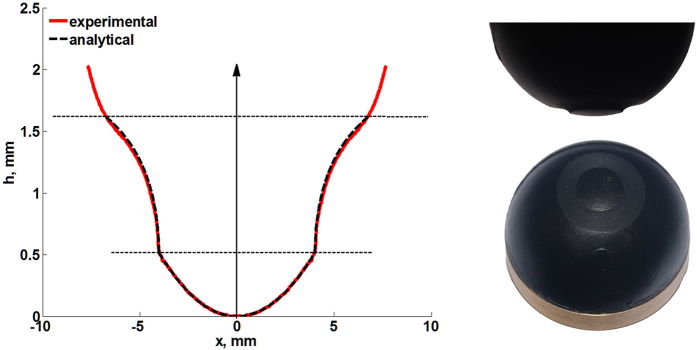
(**a**) The cross section of spherical indenter worn profile obtained experimentally due to *tangential* fretting wear. Only the final profile after a very large number of oscillation cycles is shown. The theoretical limiting worn shape calculated according to Eq. (3) for the same parameters as in the experiment is shown by the dashed curve. (**b**) Photos of the experimental indenter in the final state.

It is worth noting that even the asymptotic behaviour in the vicinity of the inner radius of the worn region coincides with the theoretical result very well. Thus, the assumption of small slopes of the surface (half-space approximation) obviously does not discredit the logic and the results of the theoretical consideration. In our opinion, such exact coincidence *even for one single fretting mode* provides sufficient support for the basic idea of the theoretical consideration and thus also for the universality of the limiting profile. In spite of this, we carried out experiments for one further fretting mode (pure tangential oscillations). Contrary to torsion, the loading in this mode is not axis-symmetrical. However, the resulting profile is axis-symmetric and its final form is described by the same eq. (3), as can be seen from Fig. 3.

### An example for the kinetics of fretting wear

We have argued that the limiting worn profile is determined solely by the radius of the permanent stick region and the maximum indentation depth during the loading cycle and doesn’t depend on the detailed history of oscillating loading or on the particular mode of oscillation. To illustrate this conclusion we carried out step-by-step simulation of the kinetics of the dual-mode wear process and compared the resulting profile with the analytical solution. We applied the rapid simulation procedure described in^19^ to an indenter which initially had a parabolic form 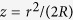, where 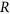 is the radius of curvature. Other than for the limiting shape, for kinetics of wear the particular form of the wear law is essential. We used the simplest law of Reye-Khrushchov-Archard^20^,^21^,^22^ stating that the linear wear is proportional to the normal or tangential stress and to the relative slip and inversely proportional to the hardness 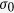, which can be written in the incremental form as


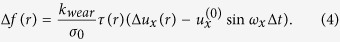


In the following we consider a dual-mode fretting under the influence of superimposed normal oscillations with amplitude 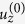 and frequency 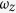 and tangential oscillations 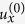 and frequency 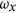 given by the relations


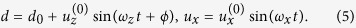


**Figure 4:**
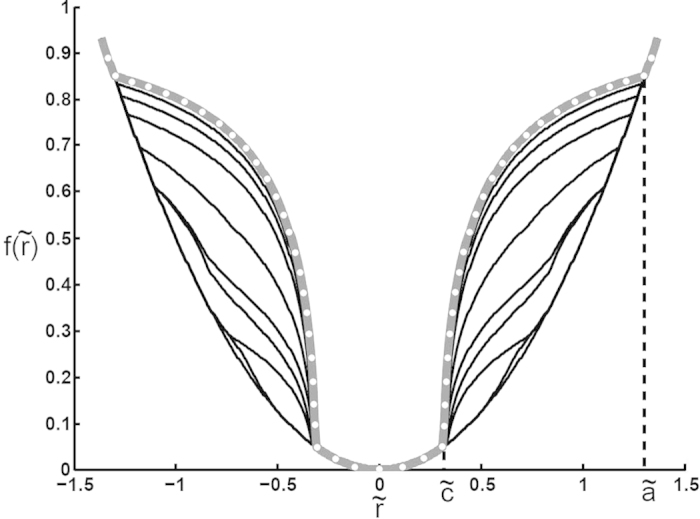
Calculated evolution of 3D profile during dual-motion fretting for the following set of parameters: *ω*_*z*_ = 0.05*ω*_*x*_, 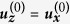
*, φ* =* 0.* The analytically calculated limiting 3D shape according to (6) is marked by bold gray curve with dots.

The maximum indentation depth, which is one of the main determining parameters of the limiting shape, is obviously given by 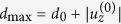. The radius of stick region is determined by Equations described in^18^ and^23^. Development of worn shape in the case when 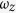 ≪ 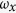 is shown in Fig. 4.

One can see complicated changes of profile due to the difference in the frequencies of normal and tangential oscillations. However, with increasing the number of oscillation cycles wear slows down and finally tends to the limiting profile shown in Fig. 4 by a bold gray line with white dots. This limiting shape is determined by the Eq. (6) (see Section “Methods”).

## Discussion

In this paper we have shown that the limiting shape due to fretting wear found in^18^ for the case of fretting due to tangential oscillations is valid in the general case of multiple-mode fretting under action of superimposed normal and tangential oscillations as well as torsion and tilting. The limiting shape does not depend on the type of fretting loading and does not depend on elastic properties of contacting bodies but only on the initial shape in the region of permanent stick, the radius of the region of permanent stick and the maximum indentation depth during the oscillation history. The universality was illustrated by a numerical example of complicated kinetics of worn profile due to normal and tangential oscillations with different frequencies and was validated by specially designed experiments with torsional and tangential fretting.

Note that the existence and the universality of the limiting worn profile suggest a simple design rule for parts subjected to oscillations: if it is known from the practice that fretting is a problem for a particular system, the parts can be brought to the limiting form during production process which guarantees further wear less running of the system. If the exact contact conditions are not known, this production step can be realized as a “running-in” procedure. The running-in can be carried out under controlled load and environmental parameters which ensure the soft wear conditions needed for development of the ideal limiting form. The environmental conditions *after* a proper running-in are then of minor importance as there is no further wear in this state.

The limiting profile described in the paper develops under very general assumptions. However even these general assumptions can still be violated under real experimental or technological conditions. One of these assumptions is the “assumption of continuous wear”. This means, that no wear particles are produced during the fretting process with a size that is comparable with the characteristic indentation depth. We have made sure that our experimental conditions meet these requirements. However, it is known that in metallic contacts these conditions can be violated due to adhesive wear. The suggested theory is not applicable to such cases. However, even in these cases, the existence of the limiting shape can be used for “soft preprocessing” under conditions for which severe wear is avoided.

## Methods

### Theory and numerical simulation

Analytical calculation of the limiting shape is based on the method of dimensionality reduction (MDR)^24^,^25^. In^18^, it was shown that the limiting worn shape, 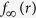, is given by


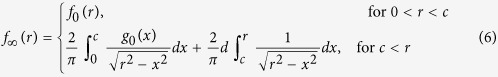


where 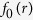 is the initial, non-worn shape of the indenter, 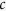 is the radius of permanent stick, 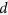 is the indentation depth (which in the case of multiple-mode fretting has to be understood as *maximum* indentation depth during the oscillation cycle, 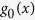 is given by the equation


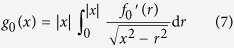


and 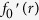 means the derivative with respect to 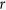. The outer radius of the worn region, 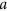, is given by the intersection of the profile (6) with the initial profile as illustrated in Fig. 1. Note that the limiting shape does depend on the initial shape, the radius of the radius of permanent stick and the maximum indentation depth but not on elastic properties of the contacting bodies.

### Numerical simulation of kinetics of fretting wear

Simulation of the progressive wear under particular double-mode fretting conditions was also carried out using the method of dimensionality reduction. The detailed description of the used method can be found in^19^.

### Experimental set up

**Figure 5:**
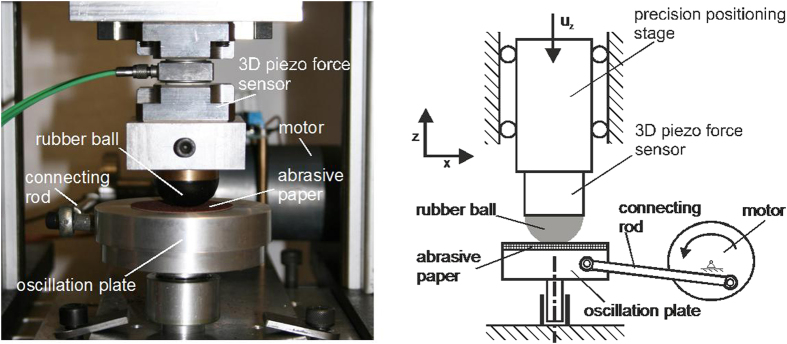


For experimental validation of the theoretically predicted universal limiting shape, an experimental set up shown in Fig. 5 was designed and built. The central working part of the setup is a rubber half-ball which was pressed against a hard abrasive surface mounted on a rotatable table. According to the theory, the limiting shape does not depend on the particular fretting mode. We therefore used the torsional mode which is the simplest for experimental realization and control. The rubber ball was indented by a given indentation depth, which remained constant during the whole experiment. After that the counter-body was rotated around the vertical axis of symmetry of the rubber specimen. After completing of 2,160 cycles, the sample was demounted and the shape was determined from photographs. Then the ball was mounted back, pressed to the same indentation depth, and loaded by torsional oscillations with the same for the next 2,160 cycles and so on. The resulting profiles with progressive wear are shown in Fig. 2. The construction and function of the experimental set up and the process of fretting wear are illustrated by a video in the supplemental material. The parameters of the experiment were: ball radius (*R*) 14.5 mm, indentation depth (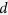) 2 mm, radius of the region of permanent stick (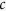) 3.25 mm. The resulting outer radius of the worn region was 7.35 mm. Other technical parameters are summarized in Table 1.

Experiments with fretting under tangential oscillations were carried out on the linear tribometer described in^26^ in which the normal force control was replaced by displacement control.

## Additional Information

**How to cite this article**: Dmitriev, A. I. *et al.* Universal limiting shape of worn profile under multiple-mode fretting conditions: theory and experimental evidence. *Sci. Rep.* **6**, 23231; doi: 10.1038/srep23231 (2016).

## Supplementary Material

Supplementary Video 1

## Figures and Tables

**Table 1:** Technical specification of the precision translation stage.

Stiffness in motion direction	[N/μm]	3500
Load capacity	[N]	200
Push/pull force	[N]	50
Lateral force	[N]	100
Resolution	[μm]	0.018
Min. incremental motion	[μm]	0.2
Backlash	[μm]	10
Unidirectional repeatability	[μm]	1
